# Quantification of intermittent retinal capillary perfusion in retinal vein occlusion and proliferative diabetic retinopathy

**DOI:** 10.1186/s40942-025-00720-2

**Published:** 2025-10-02

**Authors:** Mahadev Bhalla, Farhad Ghaseminejad, Taylor Burdett, Arman Athwal, Brendan Tao, Marinko V. Sarunic, Rony C. Preti, Eduardo V. Navajas

**Affiliations:** 1 Department of Ophthalmology and Vision Sciences, Eye Care Centre, University of British Columbia, Vancouver, BC Canada; 2 Department of Medical Physics, Biomedical Engineering, University College London, London, England; 3 Department of Ophthalmology, University of Sao Paulo, Sao Paulo, Brazil; 4 Eye Care Centre, Vancouver General Hospital, British Columbia, 2550 Willow Street, Vancouver, V5Z 3N9 Canada

**Keywords:** Optical coherence tomography angiography, Proliferative diabetic retinopathy, Retinal vein occlusion, Intermittent capillary perfusion

## Abstract

**Objective:**

To detect and quantify intermittent capillary perfusion using optical coherence tomography angiography (OCTA) in patients with branch retinal vein occlusion (BRVO), central retinal vein occlusion (CRVO), proliferative diabetic retinopathy (PDR), and healthy control eyes.

**Methods:**

OCTA images were acquired from patients with BRVO(*n* = 9), CRVO(*n* = 8), PDR(*n* = 8) and healthy controls(*n* = 10). Five 6 × 6 mm scans were registered and averaged at baseline (T0) and thirty minutes after (T30) into single en-face images of the superficial and deep vascular complexes (SVC and DVC). Pixels were labeled as vessel or non-vessel using a previously published machine learning model. Loss of Perfusion (LoP) was defined as the percentage of vessel pixels present in T0 image that disappeared at T30, and Gain of Perfusion (GoP) was defined as the percentage of vessel pixels that appeared in T30 image. The amount of intermittent capillary perfusion was the sum of GoPLoP.

**Results:**

Patients with PDR, CRVO and BRVO showed significantly higher GoPLoP values than controls in both the macular and temporal regions. The temporal region generally exhibited significantly greater GoPLoP values than the macular region. Layer analysis indicated a significantly higher GoPLoP within the DVC compared to the SVC. There was a significant negative correlation between perfusion density and perfusion variability.

**Conclusion:**

Our results demonstrate higher GoPLoP in BRVO, CRVO, and PDR patients compared to controls. This measure may be utilized as a novel biomarker of tissue hypoxia. Further studies are necessary to better elucidate the role of GoPLoP in monitoring disease progression and treatment efficacy of retinal vascular diseases.

**Supplementary Information:**

The online version contains supplementary material available at 10.1186/s40942-025-00720-2.

## Introduction

The retina is among the most metabolically active tissues in the body, yet to maintain its transparency for light perception, it possesses a limited burden of imbedded vascular structures. The retina compensates for this relative and structural blood supply limitation by functional means, denoted vasomotion, which refers to physiological oscillation of vascular tone to redirect blood flow. In the retina, vasomotion exerts local and dynamic vascular control of perfusion to the different regions of retinal tissue, according to their varying and immediate metabolic demand [1]. As a consequence, retinal perfusion is not continuous, but rather, intermittently fluctuant over periods on the order of seconds to minutes [2]. A corollary of this dynamic mechanism is that retinal regions may experience transient episodes of non-perfusion and subsequent re-perfusion, which while a normal physiological process, may be erroneously perturbed in retinal vascular diseases and predispose to retinal ischemia [1, 3]. 

Diabetic retinopathy, characterized by neurovascular degeneration secondary to chronic hyperglycemia [4], is the most common cause of vision loss from retinal vascular disease [5]. The condition may progress to proliferative diabetic retinopathy (PDR), referring to neovascularization that predispose to complications of vitreous hemorrhage, retinal tears, and tractional retinal detachment, among others [4]. Retinal vascular occlusions represent the next most common cause of vision loss from retinal vascular disease, presenting often as venous rather than arterial occlusions, known as central retinal vein occlusion (CRVO) or branch retinal vein occlusion (BRVO) [5–7]. PDR and retinal vein occlusions elicit retinal ischemia and inflammation, thereby facilitating pathologic neovascularization. The expression of several angiogenic and angio-inhibitory factors is oxygen dependent, thereby engendering abnormal neovascularization secondary to compromised perfusion. Notably, vascular endothelial growth factor (VEGF), a hypoxia-inducible cytokine, assumes a pivotal role in the genesis of these aberrant vascular formations and the ensuing visual impairment. An understanding of the mechanisms that generate functional blood flow redistribution is therefore a prerequisite for developing therapies to correct defects in blood flow control that occur after disorders such as retinal ischemia and diabetes [8]. 

Optical coherence tomography angiography (OCTA) is a non-invasive diagnostic tool which may be leveraged to evaluate retinal microvasculature perfusion dynamics and related pathology [1]. The tool detects signal changes from red blood cells in motion, as opposed to static tissues, and defers the use of contrast dye [9]. Currently, to measure perfusion and/or vessel density, most OCTA studies involve a single scan acquisition [10]. However, given the dynamic nature of retinal perfusion, for a single acquisition, it follows that capillaries that appear perfused in the initial image may no longer be perfused in subsequent imaging [10–12]. Thus, OCTA imaging in at least 2 timeframes opens the possibility to observe the phenomenon of opening and closure of capillaries known as vasomotion.

The present study sought to evaluate intermittent capillary perfusion dynamics over time via *en-face* OCTA analysis on pathological eyes (BRVO, CRVO, and PDR) and healthy controls. As vasomotion is primarily driven by tissue oxygen levels, we hypothesized a priori that the quantified intermittent capillary perfusion may be an objective biomarker for retinal vascular ischemia and capillary dropout. By extension, we hypothesized that pathological eyes would exhibit a significantly greater burden of intermittent capillary perfusion variability compared to healthy controls.

## Methods

### Study participants

This study was reviewed and approved by the institutional review board at the University of British Columbia (REB H15-02914) and abides, in full, to the principles of the Declaration of Helsinki. Subject recruitment and imaging were conducted at the Eye Care Centre (Vancouver General Hospital, Vancouver, British Columbia, Canada) between April 2021 to October 2022. All participants provided written informed consent before study participation.

Participants were included if they were aged 18 or older and deemed by the senior author (EVN) to represent one of the study groups of interest (PDR, non-ischemic CRVO, non-ischemic BRVO, or healthy control) through comprehensive clinical and imaging (fluorescein angiography, OCT, and OCA) evaluation. Exclusion criteria were: (i) media opacities preventing optimal imaging quality; (ii) active intraocular inflammation; (iii) other comorbid retinal disease (e.g., age-related macular degeneration); (iv) intraocular surgery within the 3 months preceding imaging; and (v) concurrent receipt of systemic drug therapy known to have ocular side-effects. Subjects’ medical history (type of diabetes, location of occlusion, and history of ocular surgery or procedures) and demographics were obtained through chart review.

### Image acquisition

OCTA images were acquired using the PLEXTM Elite 9000 (Carl Zeiss Meditec, Dublin, CA), a swept-source OCTA (SS-OCTA) instrument featuring a 100-kHz light source with a central wavelength of 1060 nm and a bandwidth of 100 nm. This setup provided an axial resolution of approximately 5.5 μm and a lateral resolution of about 20 μm, estimated at the retinal surface. Five 6 × 6 mm images were obtained at each retinal location (macula, temporal), and this was repeated at a baseline timepoint and 30 min after the baseline (T0, T30). Hence, twenty images total were obtained for each subject. Images were re-acquired for cases where image signal strength (as evaluated by the imaging device) was poor (< 7/10), or where motion artifact (qualitatively determined by the technician) had contaminated the image.

### Image processing

*En-face* OCTA images were generated using the built-in segmentation software on the PLEXTM Elite 9000 (Carl Zeiss Meditec, Dublin, CA) apparatus. The default segmentation settings were used to delineate the superficial vascular complex (SVC) and deep vascular complex (DVC). The SVC included the radial peripapillary capillary and superficial capillary plexus located between the inner limiting membrane and the posterior boundary of the inner plexiform layer (IPL). The DVC included the intermediate capillary plexus and deep capillary plexus from the posterior boundary of the IPL to the posterior bound of the outer plexiform layer. Projection artifacts were removed from the DVC and choriocapillaris images using the built-in PLEXTM Elite 9000 (Carl Zeiss Meditec, Dublin, CA) software (v 1.7.1.31492) before exporting. Images from the same retinal location and from each time point were then registered to one another using a customized MATLAB software (MathWorks, Inc., Natick, Massachusetts). Initial registration involved coarse translational alignment via a discrete Fourier transform, followed by precise registration using an implementation of the diffeomorphic demons algorithm. Registered images were then averaged. The averaged images from each retinal location and time point (i.e., macula-T0, macula-T30, temporal-T0, and temporal-T30) were used for the subsequent analysis.

For the quantification of retinal vessel perfusion density (PD), a previously published deep learning technique was employed to assign probabilities to each pixel, indicating whether it represented a vessel or non-vessel [13]. After segmentation, probability maps were binarized and PD was defined as the percentage of vessel-containing image area relative to the total image area.

Two flow measures were computed to assess changes in perfusion over time. Loss of Perfusion (LoP) was defined as the percentage of vessel pixels that were present in the baseline image (T0) but absent in the follow-up image (T30). Conversely, Gain of Perfusion (GoP) was defined as the percentage of vessel pixels that were present in the T30 image but not observed in the T0 image. The total burden of intermittent capillary perfusion variability across this period was calculated as the sum of the LoP and GoP, referred to herein as GoPLoP. The GoPLoP was calculated as an average over the entire image area, and additional calculated within each image quadrant (superotemporal, infratemporal, superonasal, and inferonasal).

### Statistical analysis

Demographic and clinical characteristic data are presented as mean and standard deviation. A Shapiro test revealed that all collected imaging data had significantly deviated from a normal distribution. Thus, continuous outcomes are presented as median (interquartile range; IQR) and we employ non-parametric statistics. The Kruskal-Wallis test with *post hoc* pairwise significant testing (with false detection rate correction with continuity correction for multiple comparisons; adjusted alpha of 0.05) was used to compare imaging data between study groups. Further, a Spearman’s correlation test was used to evaluate the significance and correlation strength between GoPLoP and perfusion density. Analyses and figure synthesis were conducted in R (R Core Team, R Foundation for Statistical Computing, Vienna, Austria).

## Results

We evaluated 35 eyes from 35 patients: 9 eyes in the BRVO group, 8 eyes in the CRVO group, 8 eyes in the PDR group, and 10 eyes in the control group. Table 1 depicts the demographics and pertinent clinical characteristics of included subjects.

**Table 1:** Subject demographic and clinical characteristics

	Control	BRVO	CRVO	PDR
Number of Subjects	10	9	8	8
Mean Age (years) ± SD	57.63 ± 19.85	74.33 ± 6.88	61.55 ± 15.86	52.48 ± 13.90
Gender (M/F)	5:5	5:4	1:7	5:3
Eye(s) (OD: OS)	5:5	6:3	2:6	4:4
Ethnicity (Caucasian: Non-Caucasian)	5:5	5:4	7:1	4:4
Mean Diabetes Years ± SD	-	-	-	13.83 ± 13.42
Mean A1c ± SD	-	-	8.6 (one subject)	7.93 ± 2.25
Insulin (Y/N)	0/10	2/7	1/8	2/6
Hypertension (Y/N)	0/10	9/0	5/4	4/4
Mean # of Injections ± SD	-	16.56 ± 13.99	19.75 ± 18.80	8.5 ± 10.98
Mean Injection Days Prior to Imaging ± SD	-	31.78 ± 21.25	111.25 ± 198.75	95.14 ± 87.21

Abbreviations: Proliferative Diabetic Retinopathy (PDR); Branch Retinal Vein Occlusion (BRVO); Central Vein Occlusion (CRVO); Male (M); Female (F); Right Eye (OD); Left Eye (OS); Standard Deviation (SD)

### Regional analysis of GoPLoP and PD

**Fig. 1:**
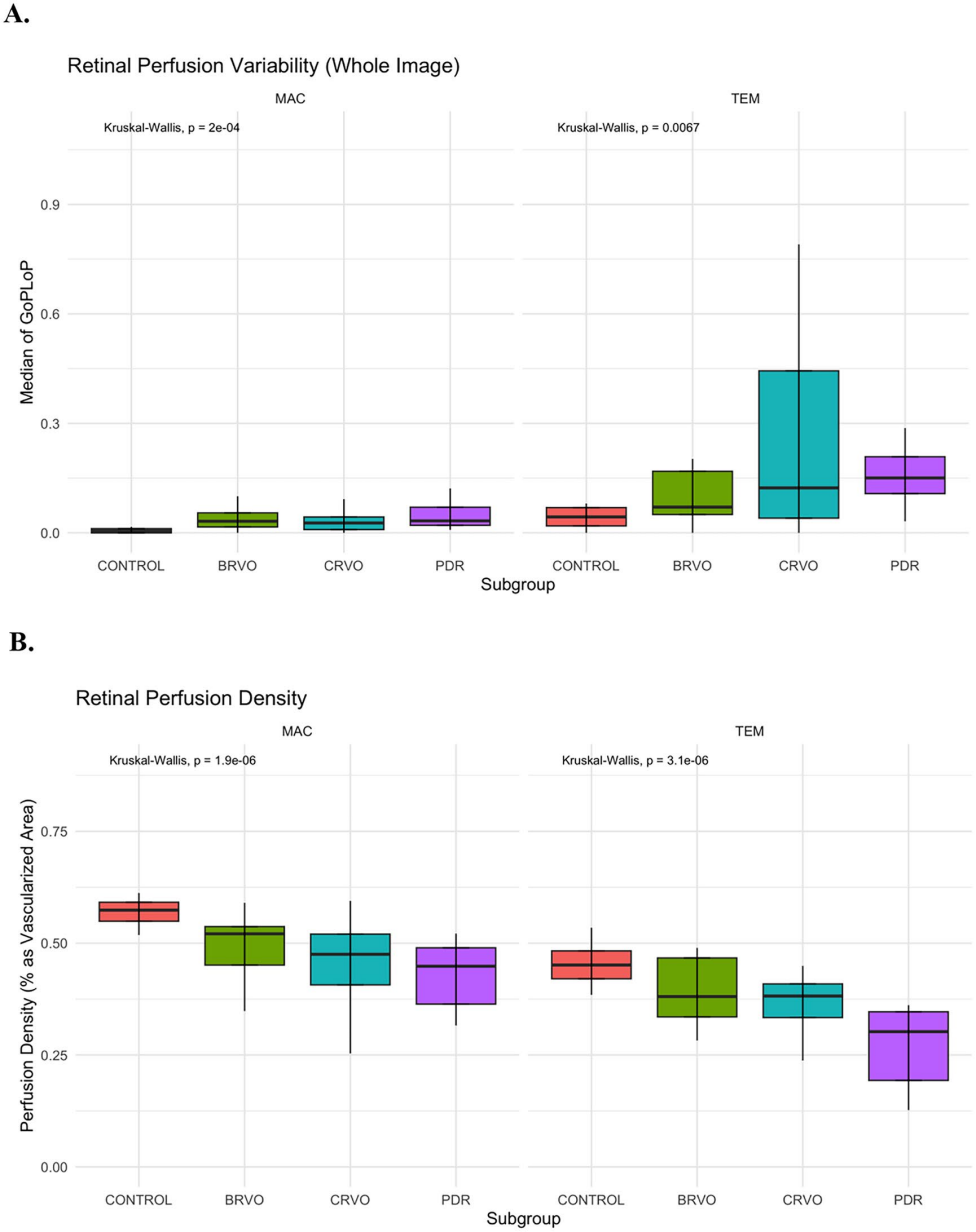
(**A**) Boxplots of retinal perfusion variability (measured as GoPLoP [gain of perfusion + loss of perfusion] on whole image ocular coherence tomography angiography over 30 min), stratified by view (macular or temporal) and study subgroup. (**B**) Boxplots of retinal perfusion density, stratified by view and study subgroup. Kruskal-Wallis and *post hoc* pairwise testing (with false detection rate adjustment for multiple comparisons) are shown (* indicates *p* < 0.05; ** indicates *p* < 0.01; ** indicates *p* < 0.001). Abbreviations: Branch Retinal Vein Occlusion (BRVO); Central Retinal Vein Occlusion (CRVO); Proliferative Diabetic Retinopathy (PDR). **Macula (MAC); Temporal (TEM); Non-significant (ns)**

Table 2 exhibits a summary of the GoPLoP and PD, stratified by retinal disease cohort, region (macular and temporal), and layer (SVC and DVC). Figure 1A illustrates the results of Kruskal-Wallis and *post hoc* pairwise analysis for retinal perfusion variability. In summary, there was a significant between-group difference in whole-image GoPLoP in the macular (*p* = 0.0002) and temporal (*p* = 0.007) views. For the macular view, *post hoc* pairwise analysis revealed that the control group had a significantly lower burden of retinal perfusion variability than the BRVO (*p* = 0.001), CRVO (*p* = 0.03), and PDR (*p* < 0.001) groups. Further, there was no between-group difference among pathological cohorts. However, for the temporal view, *post hoc* pairwise analysis only revealed that the control group had a significantly lower burden of retinal perfusion variability than the PDR group (*p* = 0.002), which was not significant albeit suggestive compared to the BRVO (*p* = 0.08) and CRVO (*p* = 0.08) groups. Nonetheless, alike the macular view, there was no between-group difference among pathological cohorts.

**Table 2:** Descriptive summary of retinal perfusion variability over time (GoPLoP = gain of perfusion + loss of perfusion over 30 min) and perfusion density. Data are stratified by retinal disease cohort, region, and layer

Cohort	Region	Layer	GoPLoP Median (IQR)	PD Median (IQR)
CRVO	Macula	DVC	0.033 (0.037)	0.43 (0.11)
		SVC	0.015 (0.021)	0.51 (0.073)
	Temporal	DVC	0.17 (0.33)	0.4 (0.057)
		SVC	0.088 (0.31)	0.35 (0.068)
BRVO	Macula	DVC	0.053 (0.042)	0.49 (0.091)
		SVC	0.031 (0.027)	0.54 (0.026)
	Temporal	DVC	0.16 (0.19)	0.42 (0.15)
		SVC	0.063 (0.031)	0.38 (0.082)
PDR	Macula	DVC	0.05 (0.075)	0.36 (0.072)
		SVC	0.031 (0.0098)	0.49 (0.059)
	Temporal	DVC	0.19 (0.052)	0.19 (0.12)
		SVC	0.11 (0.053)	0.34 (0.057)
Control	Macula	DVC	0.012 (0.013)	0.56 (0.045)
		SVC	0.0048 (0.0044)	0.58 (0.027)
	Temporal	DVC	0.053 (0.036)	0.49 (0.055)
		SVC	0.017 (0.053)	0.42 (0.035)

Abbreviations: Gain of Perfusion + Loss of Perfusion (GoPLoP); Perfusion Density (PD); Proliferative Diabetic Retinopathy (PDR); Branch Retinal Vein Occlusion (BRVO); Central Vein Occlusion (CRVO); Interquartile Range (IQR)

Supplemental Figs. 1 A-D illustrate the central tendencies and variance of retinal perfusion variability, stratified further by image quadrant. Supplemental Table 2 depicts a summary of image quadrant-level analyses of Kruskal-Wallis and *post hoc* pairwise comparisons for retinal perfusion variability. In summary, with exception of the superotemporal quadrant, the temporal view across quadrants did not exhibit significant between-cohort differences in GoPLoP. In contrast, the macular view showed at least one significant pairwise difference at each quadrant between the control and a selection of pathological cohorts. Lastly, there was significantly greater intermittent capillary perfusion in the temporal region compared to the macula, after controlling for disease, patient, and eye evaluated (*p* < 0.0001).

Figure 1B illustrates the results of Kruskal-Wallis and *post hoc* pairwise analysis for PD. In summary, there was a significant between-group difference in whole-image PD in the macular (*p* < 0.001) and temporal (*p* < 0.001) views. For the macular view, *post hoc* pairwise analysis revealed that the control group had a significantly greater PD than the BRVO (*p* = 0.0003), CRVO (*p* = 0.0003), and PDR (*p* < 0.001) groups. Further, the PDR group had had a significantly lower PD than the BRVO group (*p* = 0.02). Likewise, for the temporal view, *post hoc* pairwise analysis revealed that the control group had a significantly greater PD than the BRVO (*p* = 0.02), CRVO (*p* = 0.0004), and PDR (*p* < 0.001) groups. Further, the PDR group had had a significantly lower PD than the BRVO (*p* = 0.004) and CRVO (*p* = 0.01) group. Generally, when controlled for vascular complex layer, the temporal region had a significantly lower PD than the macular region (*p* < 0.001). As well, when controlled for regional view, the SVC had significantly lower PD than the DVC layer (*p* < 0.001).

**Fig. 2:**
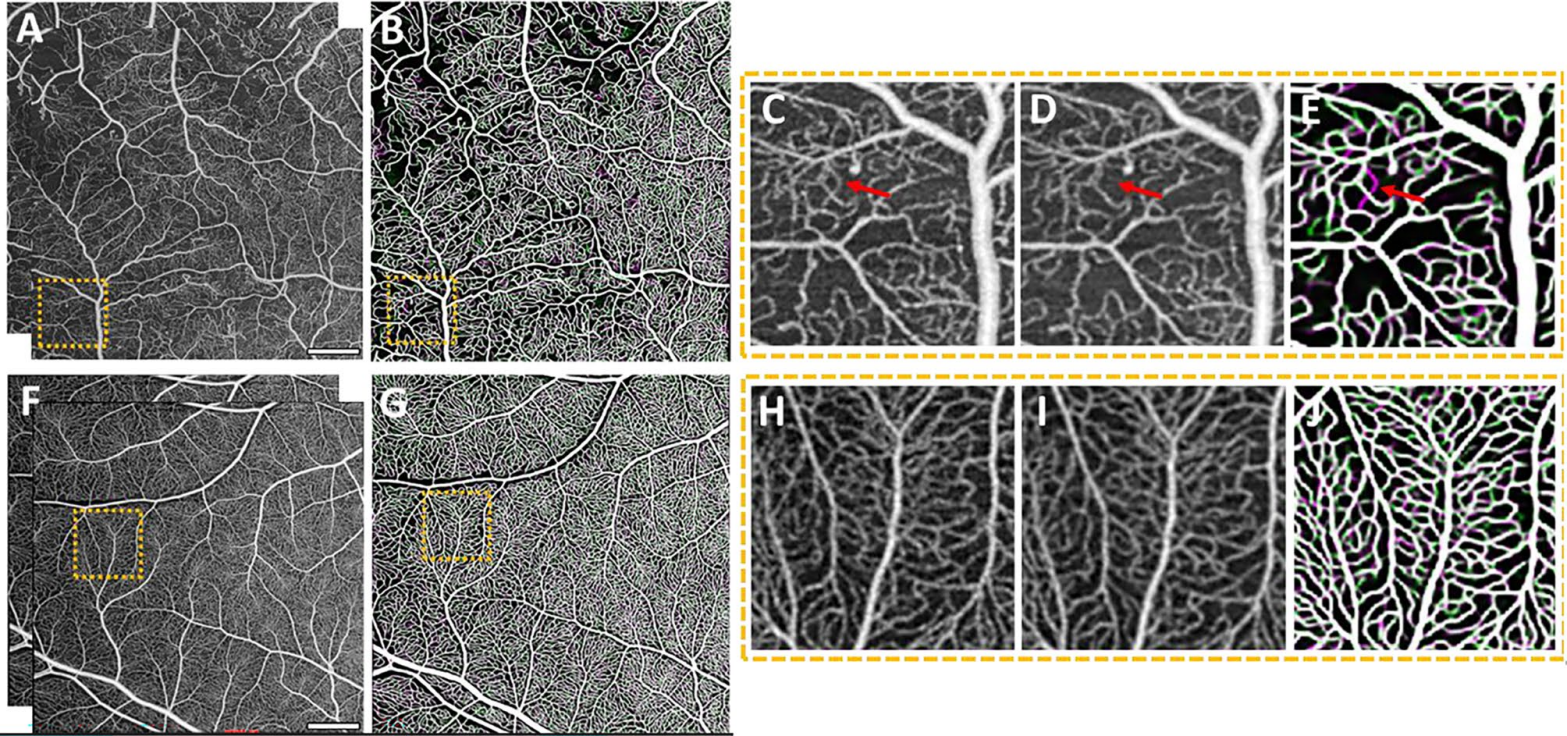
Quantification of capillary perfusion variability between two imaging sessions separated by 30 min, and comparison between a subject with PDR (top row) and a healthy control (bottom row). (**A**, **F**) Two averaged OCTA en face images acquired 30 min apart. (**B**, **G**) Neural-network generated vessel segmentation overlay of the two imaging sessions, where magenta pixels denote intermittent perfusion loss, and green pixels denote intermittent perfusion gain. (**C**-**D**, **H**-**I**) Zoomed-in windows of each imaging session’s averaged image. (**E**, **J**) Zoomed-in window of each imaging session’s vessel segmentation overlay. Red arrow in PDR images denotes a capillary that becomes un-perfused after 30 min. Scale bars = 1 mm

Qualitatively, regions of capillary perfusion and dropout over the 30-minute imaging intermission were observed amongst all groups, including the control group. Figure 2 depicts OCTA sample images that exhibit temporal capillary perfusion variability (over 30 min) in a patient with PDR (Fig. 2; top row) and a healthy control (Fig. 2; bottom row). Of note, while the control subject does exhibit minor perfusion variability, the perfusion variability in the patient with PDR is more manifest (Fig. 2; denoted by the red arrow).

### Layer analysis

**Fig. 3:**
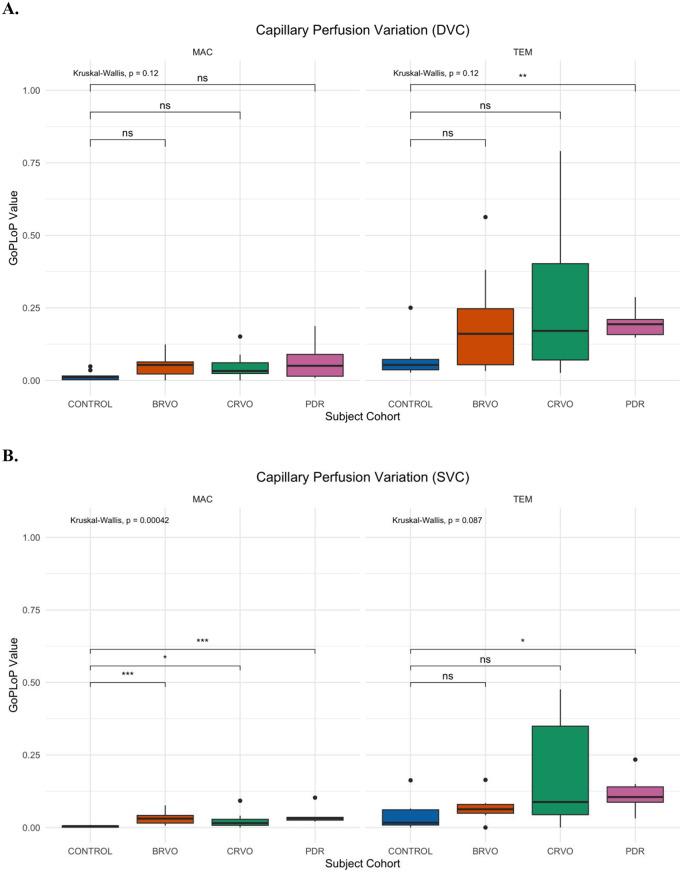
(**A**) Boxplots of retinal perfusion variability (measured as GoPLoP [gain of perfusion + loss of perfusion] on whole image ocular coherence tomography angiography over 30 min) in the deep vascular complex, stratified by view (macular or temporal) and study subgroup. (**B**) Boxplots of retinal perfusion variability (measured as GoPLoP [gain of perfusion + loss of perfusion] on whole image ocular coherence tomography angiography over 30 min) in the superficial vascular complex, stratified by view and study subgroup. Kruskal-Wallis and *post hoc* pairwise testing (with false detection rate adjustment for multiple comparisons) are shown (* indicates *p* < 0.05; ** indicates *p* < 0.01; ** indicates *p* < 0.001). Abbreviations: Deep Vascular Complex (DVC); Superficial Vascular Complex (SVC); Branch Retinal Vein Occlusion (BRVO); Central Retinal Vein Occlusion (CRVO): Proliferative Diabetic Retinopathy (PDR)

Figure 3A and B depict the GoPLoP perfusion variability of the DVC and SVC retinal layers, respectively. Across all cohorts, the DVC layer exhibited a greater burden of GoPLoP perfusion variability (*p* < 0.001).

There was no significant cohort-level difference in GoPLoP perfusion variability in the temporal nor macular DVC. There was no significant cohort-level difference in GoPLoP perfusion variability in the temporal SVC. However, within the macular SVC, the control group had a significantly lower burden of GoPLoP perfusion variability than the BRVO (*p* = 0.001) and PDR (*p* = 0.001) groups yet did not significantly differ from the CRVO group (*p* = 0.09).

### Retinal perfusion density versus perfusion variation

**Fig. 4:**
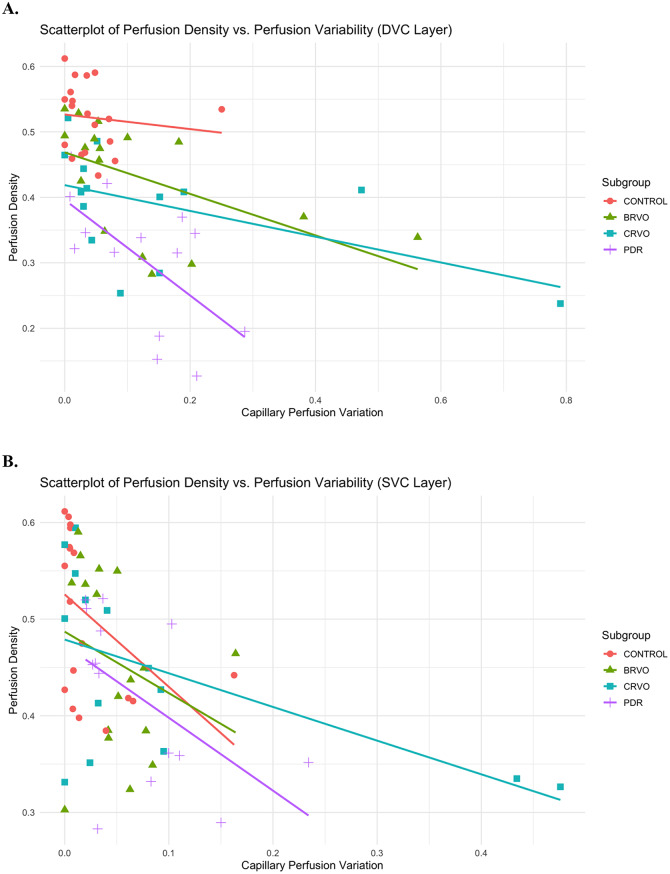
(**A**) Scatterplot of retinal perfusion density versus perfusion variability (measured as GoPLoP [gain of perfusion + loss of perfusion] on whole image ocular coherence tomography angiography over 30 min) in the deep vascular complex, stratified by study subgroup. (**B**) Scatterplot of retinal perfusion density versus perfusion variability (measured as GoPLoP [gain of perfusion + loss of perfusion] on whole image ocular coherence tomography angiography over 30 min) in the superficial vascular complex, stratified by study subgroup. Abbreviations: Deep Vascular Complex (DVC); Superficial Vascular Complex (SVC); Branch Retinal Vein Occlusion (BRVO); Central Retinal Vein Occlusion (CRVO): Proliferative Diabetic Retinopathy (PDR)

Figures 4A and B depict scatterplots of PD versus GoPLoP of the DVC and SVC retinal layers, respectively. Generally, across all subjects, retinal regions and layers, there was a qualitative negative trend between PD and GoPLoP.

## Discussion

The present study utilized GoPLoP from sequential OCTA analysis to quantify capillary perfusion heterogeneity in the macular and temporal regions of healthy controls and subjects with CRVO, BRVO, and PDR. The major findings of the study were: (i) the control group had a significantly lower burden of retinal perfusion variation than the pathological groups, which among themselves, did not demonstrate a between-group difference; (ii) the retinal perfusion variation was significantly greater within the temporal region than the macula; (iii) the retinal perfusion variation was significantly greater within the DVC compared to the SVC; and (iv) there was significant, moderate-to-strong, and negative correlation between PD and retinal perfusion variation, suggesting that more sparsely vascularized regions had greater perfusion variation.

The concept of intermittent blood flow in the retina is a crucial aspect of retinal physiology and may play a significant role in understanding various retinal vascular diseases. Intermittent blood flow refers to the pulsatile or episodic nature of blood circulation within the retinal microvasculature, including the capillaries, arterioles, and venules. The intermittent nature of blood flow in capillaries is a critical component of the local blood flow regulation and helps in meeting the metabolic demands of tissues, particularly in highly metabolic tissues like the retina. This pulsatile flow in capillaries contrasts with the continuous flow seen in larger arteries, allowing for efficient nutrient and oxygen delivery, precise regulation of blood supply, conservation of energy, waste removal, and adaptation to local conditions. The dynamic regulation of blood flow at the microvascular level ensures that tissues receive the appropriate amount of blood and nutrients precisely when they need it, contributing to tissue health and function. This phenomenon is particularly relevant in the context of conditions like diabetic retinopathy, and retinal vascular occlusions, where disruption of the normal capillary perfusion heterogeneity may aggravate the ischemic insult seen these conditions. Previous studies have suggested limited sensitivity of OCTA to blood flow velocity [14]. However, by registering and averaging five consecutive images at two different time-points in the methodology used in this study, we were able to meaningfully improve the sensitivity our assessment of flow.

We noted that the GoPLoP (perfusion variation) in all diseased groups (BRVO, CRVO, and PDR) were significantly higher than that observed in the control group. While these conditions differ in clinical presentation, underlying etiology and prognosis, a common denominator in their pathophysiology is retinal ischemia. The resultant lower oxygen concentration in areas of ischemia may lead to disruption of vasomotion, predisposing to further and more frequent episodes of dysfunctional dilation and closure of capillaries, which was captured by sequential image acquisition study design.

Another interesting finding of our study is the higher rate of intermittent capillary perfusion in the region temporal to the macula in both disease and control groups. The latter suggests that even in healthy subjects an area of the retina with lower vessel density may result in lower tissue oxygen level and higher rates of GoPLoP. The temporal area to the macula was chosen based on previous studies, demonstrating that the areas outside of the parafovea particularly temporal to the macula may be more susceptible to ischemia and may be affected early in diabetic patients [12, 15]. The temporal region to the macula was therefore chosen as a consistent target area in the peripheral retina in all subjects, regardless of the disease group or the affected region of the retina. This was done in order to preclude any selection bias. We believe that imaging the macula as well as the peripheral retina is important in our understanding of how retinal tissue as a whole respond to ischemic disease.

Our results indicated significantly higher capillary blood flow variation in the DVC compared to the SVC. The DVC supplies the inner retinal layers and is more prone to microvascular changes and metabolic stress, leading to more pronounced variations in blood flow. This finding is corroborated by studies indicating that the DVC is more affected by ischemic conditions and has a greater role in retinal health and disease progression than the SVC [16, 17].

Finally, we observed a qualitative negative trend between GoPLoP and PD, which was even observed within healthy eyes, suggesting that intermittent capillary perfusion may provide an indirect measure of tissue oxygen levels before retinal ischemia ensues. Previous research has shown the correlation between an increase in capillary non-perfusion areas and diseases like glaucoma [18, 19] DR [12, 13] and hypertension [20]. Additionally, it has been shown that an increase in capillary non-perfusion is correlated with disease stage in DR [12]. Therefore, our findings may imply that intermittent capillary perfusion could also be a strong marker of disease progression and at an earlier stage than capillary non perfusion. This warrants further exploration by a prospective study with larger subject base.

Limitations to this study warrant further discussion. First, the sample size for each group was small in this pilot study, and given this, future studies with larger sample sizes are encouraged. Second, we did not conduct measurement of tissue oxygen levels, which in future works, may be used to correlate with regions of high perfusion variability and low perfusion density. Third, glycemic control status, duration, and stage of disease were not recorded for the PDR patients, factors that may be considered as confounds in the level perfusion variation. Lastly, recent studies have demonstrated the possible role of intraocular pressure in intermittent retinal capillary perfusion [21, 22] a variable that was not considered in our study. We note that the utilized scanning protocol in this study, which required several OCTA scans at each retinal view over two sittings (30 min apart) to calculate averaged images, may greatly limit current clinical implementation due to workflow and patient-level (e.g., ability to have stable fixation and tolerate longer acquisition time) constraints. Future studies should attempt to optimize the image acquisition workflow efficiency (e.g., whether fewer scans could be used to generate sufficient averaged images) of the present analysis, which could increase clinical utility.

## Conclusion

Our results highlight significant GoPLoP in BRVO, CRVO, and PDR patients compared to controls, and this novel measure may be a candidate biomarker for capillary perfusion heterogeneity disturbances secondary to low tissue oxygen levels. This study lends further support to the suggestion that monitoring intermittent capillary perfusion in patients with retinal vascular diseases could provide utility in monitoring of the disease and in evaluating treatment efficacy.

## Supplementary Information

Below is the link to the electronic supplementary material.

Supplementary Material 1

## Data Availability

No datasets were generated or analysed during the current study.
