# Supplementary material for: Quantification of intermittent retinal capillary perfusion in retinal vein occlusion and proliferative diabetic retinopathy
Source: Int J Retina Vitreous. 2025 Oct 2;11:102. doi: 10.1186/s40942-025-00720-2 (PMC12492569; doi:10.1186/s40942-025-00720-2)
Supplement: Supplementary file 1 — Supplementary Material 1 [file 40942_2025_720_MOESM1_ESM.docx]

**Supplemental Table 1**: Quadrant-stratified summary of retinal perfusion variability over time (GoPLoP = gain of perfusion + loss of perfusion over 30 minutes) and perfusion density. Data are also stratified by retinal disease cohort, region, and layer. Abbreviations: Gain of Perfusion + Loss of Perfusion (GoPLop); Perfusion Density (PD); Proliferative Diabetic Retinopathy (PDR); Branch Retinal Vein Occlusion (BRVO); Central Vein Occlusion (CRVO); Interquartile Range (IQR).

|  |  |  | **GoPLoP Median (IQR)** | | | |
| --- | --- | --- | --- | --- | --- | --- |
| **Cohort** | **Region** | **Layer** | **Supertemporal** | **Superonasal** | **Inferotemporal** | **Inferonasal** |
| CRVO | Macula | DVC | 0.091 (0.086) | 0.025 (0.032) | 0.049 (0.13) | 0.013 (0.048) |
|  |  | SVC | 0.028 (0.05) | 0.011 (0.052) | 0.013 (0.04) | 0.011 (0.024) |
|  | Temporal | DVC | 0.25 (0.56) | 0.081 (0.17) | 0.4 (0.48) | 0.065 (0.22) |
|  |  | SVC | 0.38 (0.56) | 0.038 (0.14) | 0.031 (0.53) | 0.054 (0.15) |
| BRVO | Macula | DVC | 0 (0.063) | 0.059 (0.16) | 0.022 (0.06) | 0.026 (0.066) |
|  |  | SVC | 0 (0.037) | 0.048 (0.08) | 0.022 (0.033) | 0 (0.021) |
|  | Temporal | DVC | 0.18 (0.22) | 0.25 (0.63) | 0.086 (0.1) | 0.14 (0.096) |
|  |  | SVC | 0.02 (0.13) | 0.048 (0.14) | 0.035 (0.13) | 0.0059 (0.025) |
| PDR | Macula | DVC | 0.07 (0.14) | 0 (0.03) | 0.062 (0.15) | 0.046 (0.056) |
|  |  | SVC | 0.024 (0.077) | 0.053 (0.041) | 0.036 (0.052) | 0.026 (0.039) |
|  | Temporal | DVC | 0.24 (0.29) | 0.44 (0.5) | 0.13 (0.11) | 0.19 (0.21) |
|  |  | SVC | 0.046 (0.19) | 0.15 (0.089) | 0.12 (0.23) | 0.11 (0.23) |
| Control | Macula | DVC | 0 (0.024) | 0 (0.016) | 0 (0) | 0 (0.04) |
|  |  | SVC | 0 (0.023) | 0 (0) | 0 (0) | 0 (0) |
|  | Temporal | DVC | 0.029 (0.023) | 0.057 (0.1) | 0.096 (0.15) | 0.05 (0.056) |
|  |  | SVC | 0 (0) | 0.027 (0.12) | 0 (0.04) | 0 (0.041) |

**Supplemental Table 2**: Kruskal-Wallis (KW) and *post hoc* pairwise testing (with false detection rate correction for multiple comparisons) of retinal perfusion variability of retina image quadrants between study cohorts. Significant values are denoted in red font.

| **Macula** | | | |
| --- | --- | --- | --- |
| **Superotemporal** |  |  |  |
| KW: 0.03 | *BRVO* | *CONTROL* | *CRVO* |
| *CONTROL* | 0.55 |  |  |
| *CRVO* | 0.19 | 0.035 |  |
| *PDR* | 0.29 | 0.099 | 0.85 |
| **Superonasal** |  |  |  |
| KW: 0.0034 | *BRVO* | *CONTROL* | *CRVO* |
| CONTROL | 0.0048 |  |  |
| CRVO | 0.11 | 0.05 |  |
| PDR | 0.42 | 0.026 | 0.42 |
| **Inferotemporal** |  |  |  |
| KW: 0.001 | *BRVO* | *CONTROL* | *CRVO* |
| *CONTROL* | 0.0081 |  |  |
| *CRVO* | 0.72 | 0.0081 |  |
| *PDR* | 0.18 | 0.00079 | 0.49 |
| **Inferonasal** |  |  |  |
| KW: 0.036 | *BRVO* | *CONTROL* | *CRVO* |
| *CONTROL* | 0.21 |  |  |
| *CRVO* | 0.87 | 0.21 |  |
| *PDR* | 0.21 | 0.028 | 0.21 |
| **Temporal** | | | |
| **Superotemporal** |  |  |  |
| KW: 0.0064 | *BRVO* | *CONTROL* | *CRVO* |
| *CONTROL* | 0.18 |  |  |
| *CRVO* | 0.19 | 0.019 |  |
| *PDR* | 0.31 | 0.021 | 0.26 |
| **Superonasal** |  |  |  |
| KW: 0.11 | *BRVO* | *CONTROL* | *CRVO* |
| *CONTROL* | 0.3 |  |  |
| *CRVO* | 0.47 | 0.57 |  |
| *PDR* | 0.57 | 0.15 | 0.31 |
| **Inferotemporal** |  |  |  |
| KW: 0.11 | *BRVO* | *CONTROL* | *CRVO* |
| *CONTROL* | 0.24 |  |  |
| *CRVO* | 0.48 | 0.24 |  |
| *PDR* | 0.64 | 0.24 | 0.48 |
| **Inferonasal** |  |  |  |
| KW: 0.55 | *BRVO* | *CONTROL* | *CRVO* |
| *CONTROL* | 0.93 |  |  |
| *CRVO* | 0.65 | 0.65 |  |
| *PDR* | 0.65 | 0.65 | 0.93 |


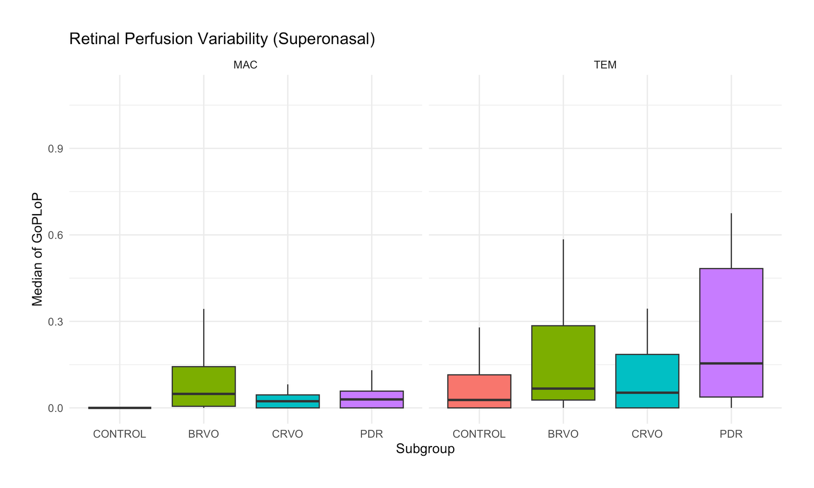

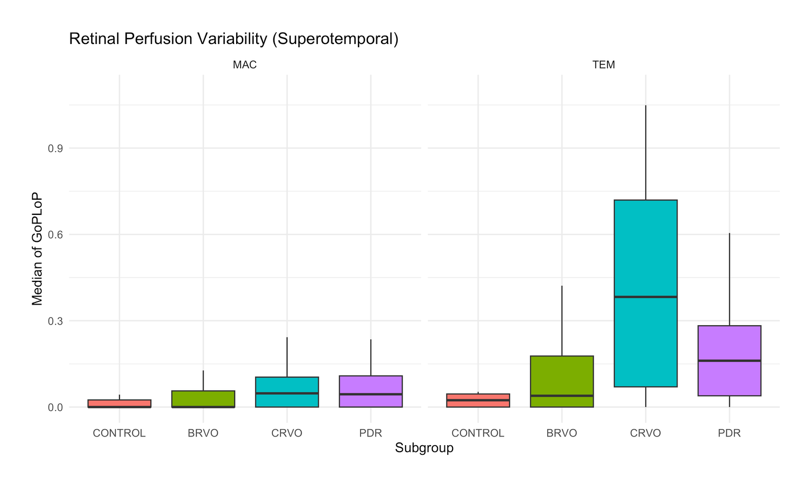


**B**

**A**


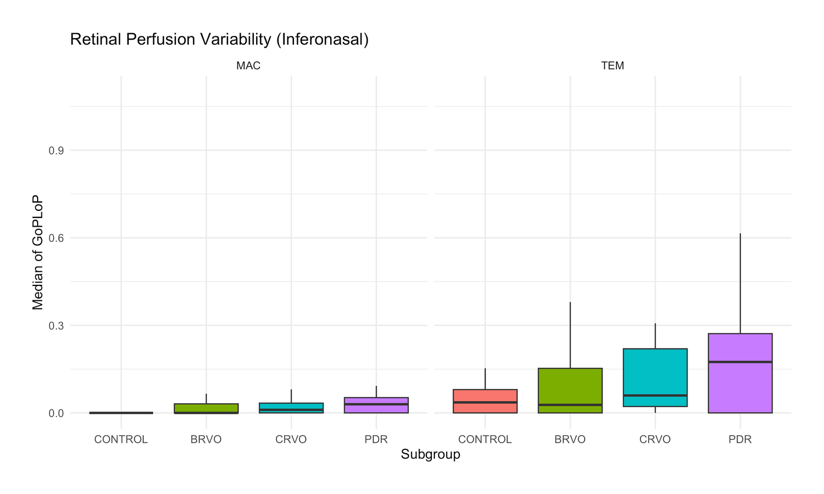

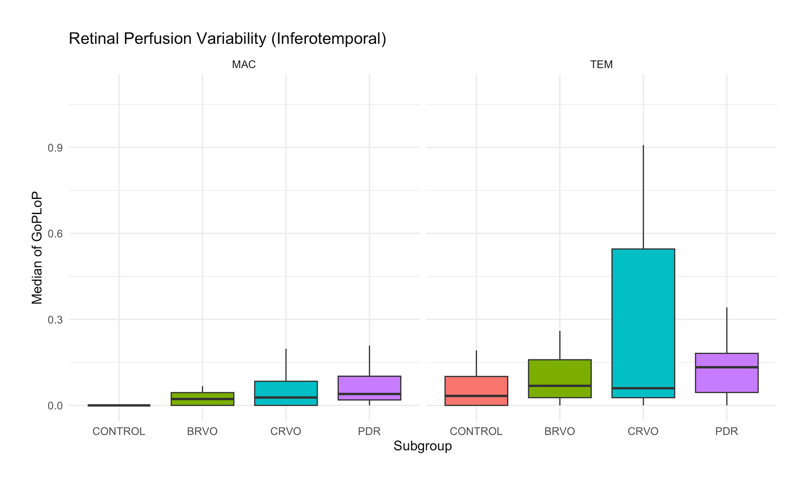


**D**

**C**

**Supplemental Figure 1**: (A) Boxplots of superotemporal quadrant retinal perfusion variability (measured as GoPLoP [gain of perfusion + loss of perfusion] on ocular coherence tomography angiography over 30 minutes), stratified by view (macular or temporal) and study subgroup. (B) Boxplots of superonasal quadrant retinal perfusion variability (measured as GoPLoP [gain of perfusion + loss of perfusion] on ocular coherence tomography angiography over 30 minutes), stratified by view (macular or temporal) and study subgroup. (C) Boxplots of inferotemporal quadrant retinal perfusion variability (measured as GoPLoP [gain of perfusion + loss of perfusion] on ocular coherence tomography angiography over 30 minutes), stratified by view (macular or temporal) and study subgroup. (D) Boxplots of inferonasal quadrant retinal perfusion variability (measured as GoPLoP [gain of perfusion + loss of perfusion] on ocular coherence tomography angiography over 30 minutes), stratified by view (macular or temporal) and study subgroup. Abbreviations: Branch Retinal Vein Occlusion (BRVO); Central Retinal Vein Occlusion (CRVO): Proliferative Diabetic Retinopathy (PDR).
